# 1868. Epidemiology of Healthcare Facility-Associated Nontuberculous Mycobacteria at a 10-Hospital Network

**DOI:** 10.1093/ofid/ofad500.1696

**Published:** 2023-11-27

**Authors:** Arthur W Baker, Ricardo M La Hoz, Judith A Anesi, Jennie H Kwon, Anastasia Wasylyshyn, Emily S Ford, Susan Harrington, Melissa B Miller, David J Weber, Emily Sickbert-Bennett, Thomas R Talbot, M Hong Nguyen, Kailey Hughes Kramer, Katelin B Nickel, Ahmed Maged, Salah Haridy, Barbara D Alexander, Jason E Stout, Deverick J Anderson

**Affiliations:** Duke University School of Medicine, Durham, North Carolina; University of Texas Southwestern Medical Center, Dallas, TX; University of Pennsylvania Perelman School of Medicine, Philadelphia, PA; Washington University - School of Medicine, St. Louis, MO; University of Michigan, Ann Arbor, MI; University of Washington, Seattle, Washington; Cleveland Clinic, Cleveland, Ohio; University of North Carolina School of Medicine, Chapel Hill, NC; University of North Carolina, Chapel Hill, NC; UNC Medical Center, Chapel Hill, North Carolina; Vanderbilt University Medical Center, Nashville, Tennessee; University of Pittsburgh School of Medicine, Pittsburgh, Pennsylvania; Division of Infectious Diseases, Department of Medicine, University of Pittsburgh School of Medicine, Pittsburgh, Pennsylvania; Washington University in St. Louis, Saint Louis, Missouri; City University of Hong Kong, Kowloon, Not Applicable, Hong Kong; University of Sharjah, Sharjah, Sharjah, United Arab Emirates; Duke University School of Medicine, Durham, North Carolina; Duke University School of Medicine, Durham, North Carolina; Duke Center for Antimicrobial Stewardship and Infection Prevention, Durham, North Carolina

## Abstract

**Background:**

Numerous studies have reported that rates of nontuberculous mycobacteria (NTM) infections are increasing. However, data on the epidemiology of healthcare facility-associated (HCFA) NTM are sparse. We performed a multicenter longitudinal study to analyze the epidemiology of NTM at a network of U.S. academic hospitals.

**Methods:**

We retrospectively analyzed data on positive cultures for NTM obtained from 2012-2020 at a network of 10 U.S. academic hospitals and associated clinics (Table). Variables analyzed included NTM species, specimen source, and hospital admission status. A unique NTM episode was defined as a patient’s first positive culture for a particular NTM species and specimen source category (pulmonary vs. extrapulmonary). Episodes linked to isolates obtained on day 3 or later of hospitalization were considered to represent hospital-onset (HO) NTM. Seven hospitals contributed at least 12 months of baseline data prior to January 2014, and within this closed cohort, trends of NTM incidence rates were estimated with log regression.

**Figure:**
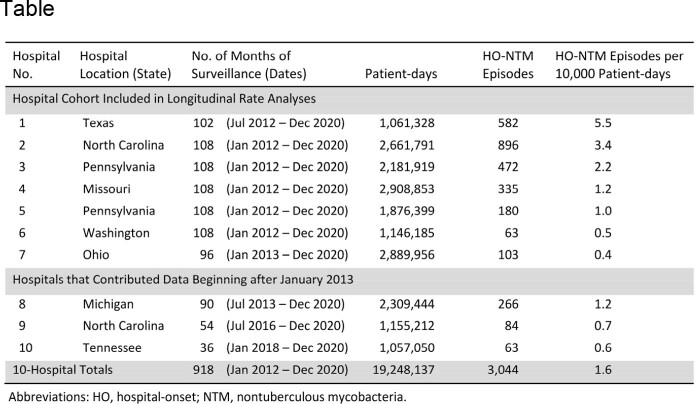

Characteristics of a 10-hospital network that performed retrospective culture-based NTM surveillance from 2012-2020.

**Results:**

Across the 10-hospital network, 24,376 total NTM isolates were identified during 19,248,137 patient-days of surveillance; 12,847 (53%) isolates represented unique NTM episodes. Of these episodes, 3,044 (24%) were HO-NTM, which were most commonly caused by *M. avium* complex (n=1,466, 48%), *M. abscessus* complex (n=397, 13%), and *M. chelonae*-*M. immunogenum* (n=348, 11%). For 595 (20%) HO episodes, specimen source was extrapulmonary.

Individual hospital incidence rates of HO-NTM were highly variable with a median rate of 1.1 episodes per 10,000 patient-days (range, 0.4 – 5.5 episodes) (Table). For the 7-hospital closed cohort, the HO-NTM incidence rate decreased from 2.3 to 1.4 episodes per 10,000 patient-days from 2014 to 2020 (incidence rate ratio, 0.6; 95% CI, 0.5-0.7; P < .0001) (Figure 1). Trend analysis estimated that the rate of HO-NTM decreased by 10% per year (95% CI, 8-12%; P < .0001) (Figure 2).

**Figure:**
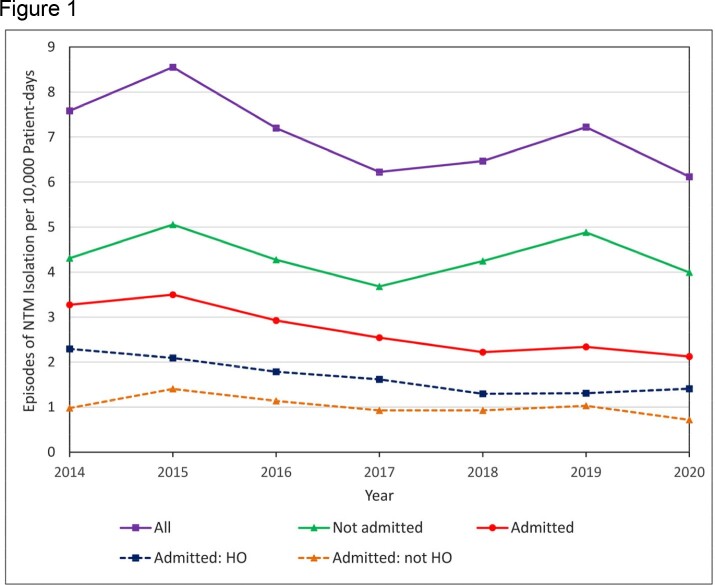

Incidence rates of NTM episodes observed from 2014-2020 at a 7-hospital cohort. Admitted epsiodes consisted of episodes that were hospital-onset (HO) or not HO.

**Figure:**
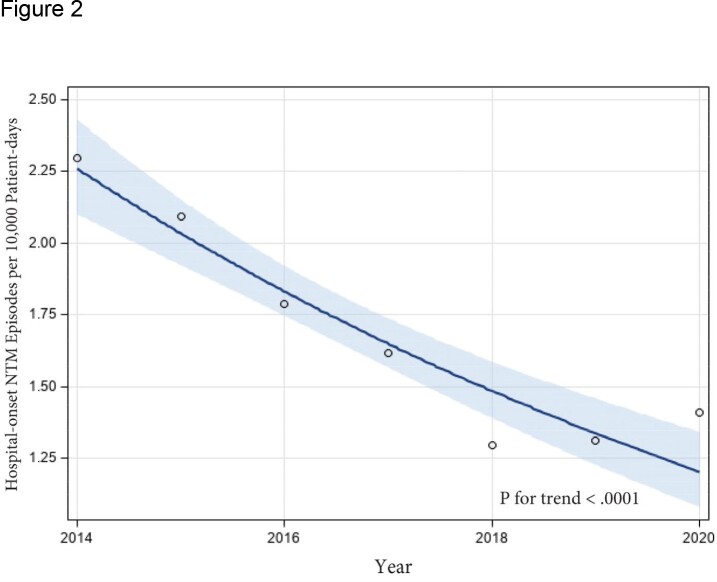

Log regression model of hospital-onset NTM incidence rates from 2014-2020 within a 7-hospital cohort. The fit plot displays predicted values with 95% confidence limits and observed rates.

**Conclusion:**

Network HO-NTM incidence rates decreased from 2014-2020, but rates varied substantially at individual hospitals. These results provide comprehensive data on HCFA-NTM isolation, including rates that can serve as external benchmarks. Given hospital variability, NTM surveillance at the individual hospital level is paramount.

**Disclosures:**

**Arthur W. Baker, MD, MPH**, Insmed: Grant/Research Support|Medincell: Advisor/Consultant **Ricardo M. La Hoz, MD**, Takeda: Advisor/Consultant **Melissa B. Miller, PhD**, BioFire: Advisor/Consultant|BioFire: Honoraria|Cantata Bio: Grant/Research Support|Luminex Molecular Diagnostics: Advisor/Consultant|Luminex Molecular Diagnostics: Honoraria|MiraVista Diagnostics: Advisor/Consultant|MiraVista Diagnostics: Honoraria|QIAGEN: Advisor/Consultant|QIAGEN: Grant/Research Support|QIAGEN: Honoraria **David J. Weber, MD, MPH**, BD: Advisor/Consultant|Germitic: Advisor/Consultant|GSK: DSMB|PDI: Advisor/Consultant|Pfizer: Advisor/Consultant|Wellair: Advisor/Consultant **Barbara D. Alexander, MD**, F2G Pharmaceuticals: Advisor/Consultant|HealthTrackRx: Advisor/Consultant|HealthTrackRx: Board Member|Leadiaint: Grant/Research Support|Merck: Advisor/Consultant|Scynexis: Grant/Research Support|Thermofisher: Advisor/Consultant **Jason E. Stout, MD, MHS**, AN2 pharmaceuticals: Grant/Research Support

